# Transcriptome Changes Driving Multiple Regulatory Pathways Involved in TGF-β-Induced Anterior Subcapsular Cataract

**DOI:** 10.3390/cells15141263

**Published:** 2026-07-14

**Authors:** Sarah Y. Coomson, Chirag Parsania, Charles G. Bailey, Cynthia Metierre, Mary Flokis, Salil A. Lachke, Frank J. Lovicu

**Affiliations:** 1 Department of Biological Sciences, University of Delaware, Newark, DE 19716, USA; sycooms@udel.edu (S.Y.C.);; 2 Cancer & Gene Regulation Laboratory, Centenary Institute, Sydney, NSW 2006, Australia; c.parsania@centenary.org.au (C.P.); c.bailey@centenary.org.au (C.G.B.); c.metierre@centenary.org.au (C.M.); 3 School of Medical Sciences, Faculty of Medicine and Health, The University of Sydney, Sydney, NSW 2006, Australia; mary.flokis@sydney.edu.au; 4 Center for Bioinformatics and Computational Biology, University of Delaware, Newark, DE 19716, USA; 5 Save Sight Institute, Faculty of Medicine and Health, The University of Sydney, Sydney, NSW 2006, Australia

**Keywords:** TGF-β1, cataract, lens, transcriptome, epithelium, epithelial–mesenchymal transition (EMT), endoplasmic reticulum (ER) stress

## Abstract

Transforming Growth Factor-beta (TGF-β) promotes lens epithelial–mesenchymal transition (EMT) and fibrosis, contributing to anterior subcapsular cataract (ASC) formation. Transgenic mice overexpressing *TGF-β1* in the lens have been studied for over three decades, and yet the impact of active *TGF-β1*-overexpression on the lens epithelial transcriptome is undefined. We have addressed this knowledge gap by examining the gene expression landscape of these unique lens epithelia. High-throughput RNA-sequencing was performed on isolated lens epithelia from three-week-old *TGF-β1*-overexpression transgenic mice from two independent lines, OVE853 and OVE918, and wild-type mice. Downstream analyses included comparisons with lens datasets (e.g., cataract surgery model) and investigations using various resources/tools (e.g., Gene Ontology, CompBio, and iSyTE). Compared to wild-type murine lens epithelia, 384 differentially expressed genes (DEGs) were commonly identified in the lens of both transgenic lines. Candidates involved in EMT, inflammatory response, extracellular matrix organization, and mechano-sensation were elevated, while those involved in lipid metabolism, Wnt-suppression, Bmp- and Notch-activation were reduced. Comparative analyses with temporal transcriptomes on a mouse cataract surgery model identified overlapping pathological pathways, and some elevated genes, for example, endoplasmic reticulum stress genes, were consistent with human ASC data. This study provides the first comprehensive transcriptomic characterization of two independent TGF-β1 transgenic ASC models and identifies novel candidate downstream genes and pathways associated with TGF-β1 overexpression. All our data is made user-friendly and accessible through iSyTE.

## 1. Introduction

Human anterior subcapsular cataract (ASC) presents as a focal lens opacity characterized as a central fibrotic plaque beneath the anterior lens capsule. It can result from ocular trauma, ocular surgery, inflammation, long-term medications, and is also linked to atopic dermatitis [1]. ASC is derived through a distinct transformation of lens epithelial cells that undergo an epithelial-to-mesenchymal transition (EMT), where the epithelial cell monolayer is destabilized to lose its epithelial traits as cells transform into myofibroblasts [2,3]. These transformed cells secrete excessive amounts of extracellular matrix (ECM) and acquire migratory and contractile properties through the accumulation of alpha-smooth muscle actin and tropomyosin [4,5], allowing them to modify the overlying lens capsule with this fibrotic plaque, which is commonly associated with capsular “wrinkling”.

Transforming Growth Factor-beta (TGF-β) is a well-documented driver of ASC [6,7], along with inflammatory cytokines and enzymes, such as matrix metalloproteinases (MMPs) [8], which contribute to lens capsule remodeling and facilitate the migration of the transformed cells. Research by multiple laboratories over the years has focused on understanding the formation of fibrotic cataracts, particularly the inherent EMT process, using a variety of in vitro and in situ models. The findings that ASC shares common mechanisms with another form of cataract, posterior capsule opacification (PCO, also referred to as secondary cataract), resulting from post-cataract surgical complications, have seen a large impetus in exploring the underlying molecular and cellular properties of EMT [9].

Researchers have adopted different models to emulate the EMT associated with the complex pathophysiology of human ASC and PCO, from immortalized lens epithelial cell lines [10], lens epithelial explants and ex vivo cultured whole lens, to in situ lens wounding models (including a cataract surgery mouse model), as well as more complex transgenic mouse models that result in fibrotic plaque formation, as utilized in the current study [11].

In vitro studies using cultured lens cells have provided many important insights into cataract formation, from the initial identification of TGF-β as the primary inducer of EMT [1,5,12,13] to understanding the cellular and molecular changes involved in this process, including its regulation by characterizing the multiple integrated signaling pathways [14,15]. While “native” human lens epithelial explants are more clinically relevant for studies, their limited numbers and finite lifespan are a limiting factor, as is the case for immortalized human lens epithelial cell lines and three-dimensional (3D) lens organoids. In contrast, in vivo animal models, albeit more expensive, allow the study of the onset and long-term maturation of cataracts in their native ocular environment (e.g., bathed by aqueous). In situ models, compromising the blood-aqueous barrier, including injury-induced EMT to the murine lens that emulate traumatic cataract, similar to ASC, and mock-cataract surgery leading to EMT associated with PCO, are dependent on technical consistency and have also provided many key molecular targets for intervention.

Mice engineered to overexpress a self-activating form of human TGF-β1, specifically in the lens, under the influence of a modified alpha-crystallin promoter, are considered the “gold standard” in situ model for mechanistic studies into fibrotic cataract formation [6,16]. These transgenic mouse lines allow researchers to follow the onset and development of anterior subcapsular plaques that are histologically identical to human ASC [6]. While comparative proteomic studies on human ASC with rodents mimic the “core” fibrotic program, the more acute injury-based and transgenic rodent studies differ in protein modifications (e.g., deamidation of crystallins) and metabolic signatures (e.g., oxidative damage) that may take many years in humans. Despite these limitations and the fact that the transgenic lines have already been well-characterized at the cellular and molecular level, there is a knowledge gap regarding the transcriptomic alterations associated with this in situ ASC pathology in both human and animal models.

Here, we present the first detailed high-resolution transcriptome analyses of the mature TGF-β1-induced pathological changes that form in two independent TGF-β1-overexpression transgenic mouse lines (OVE853, OVE918) [16]. Our findings identify the gene expression changes linked to EMT, inflammatory response, and ECM remodeling, which are hallmarks of human ASC. Moreover, our study also uncovers a role for dysregulation of lipid metabolism and endoplasmic reticulum (ER) stress in TGF-β1-induced pathology, like that observed in human ASC, suggesting the utility of these mouse transgenic models for human ASC studies. Furthermore, these data identify other pathways and genes that are associated with loss of epithelial character (e.g., reduced BMP-signaling, reduced specific epithelial gene expression, reduced lipid metabolism, etc.) as well as new high-priority downstream genes of the TGF-β1 pathways, which may represent candidates for future investigations and therapeutic interventions.

## 2. Materials and Methods

### 2.1. Mouse Studies

All experimental procedures conformed to the Association for Research in Vision and Ophthalmology (ARVO) statement for the use of animals in ophthalmic and vision research and were approved by the University of Sydney animal ethics committee. Transgenic mice at three weeks of age, from families OVE853 and OVE918 on an FVB/N background (initially described by Srinivasan and colleagues [16]), were used for the present study and were compared to wild-type (WT) mice of the same background. The transgenic lines express a secreted, constitutively active form of human TGF-β1 under the control of the lens-specific murine αA-crystallin promoter. These lines develop ASC, and, as mentioned, have been extensively characterized [6,16]. For each of the different groupings, up to 28 inbred, homozygous weaner mice were assayed. To ensure biological variation and minimize any litter-specific bias, animals were randomly selected from different batches of different litters, with an equal distribution of males and females.

For OVE853 mice, we collected 3 samples for RNA extraction and sequencing, with each sample containing up to 18 freshly isolated lens epithelial explants from 9 mice containing an anterior subcapsular plaque. Similarly, for OVE918 mice, we collected 4 samples, with each sample containing up to 14 freshly isolated lens epithelial explants from 7 mice containing an anterior subcapsular plaque. For comparison, we collected 3 samples of lens epithelia for RNA extraction and sequencing from up to 9 WT mice per sample. All tissues for each sample were immediately pooled in cold 500 µL Trizol reagent and stored at −80 °C for subsequent RNA extraction. RNA extraction from lens epithelial explants was performed according to the manufacturer’s instructions. Total RNA (2.5 µg) was DNase-treated using the TURBO DNA-*free* Kit (Invitrogen, VIC, Australia) as per the manufacturer’s protocol. RNA quantity and quality were determined using the RNA 6000 Nano kit on a Bioanalyzer (Agilent Technologies, VIC, Australia). All RNA samples with a RIN value of >7.0 were sent for mRNA sequencing (150 bp paired end, Illumina) at the Australian Genome Research Facility.

### 2.2. RNA-Seq Data Processing, Visualization, and Differential Expression Analysis

RNA-seq data were processed similarly to the previous analysis [17]. Briefly, paired-end RNA sequencing libraries (150 bp read length) were processed using a standardized computational workflow. Raw reads were trimmed to remove sequencing adapters and low-quality bases using Cutadapt (v5.2) [18]. Trimmed reads were aligned to the mouse reference genome (GRCm39) using STAR (v2.7.11b) [19]. Aligned reads were quantified at the gene level using featureCounts (v2.0.8) [20] with reverse-stranded read assignment. Per-sample RNA integrity (RIN), sequencing depth, and mapping statistics are provided in Supplementary Table S1. Gene expression values were calculated as fragments per kilobase of transcript per million mapped reads (FPKM) by normalizing raw counts to both gene length and library size. Gene lengths were determined from the NCBI RefSeq genome annotation (mm39.ncbiRefSeq.gtf) by the sum of the exonic sequences and matched to the count matrix by gene identifiers. Genes with expression ≥ 2 FPKM in at least half of all samples were retained and subjected to downstream differential expression analysis. The corresponding raw count matrix was then subset for differential expression analysis. Normalization was performed using the trimmed mean of M-values (TMM) method in edgeR via the calcNormFactors function [21]. A no-intercept design matrix was generated using model.matrix (~0 + group), with separate columns corresponding to WT, OVE853, and OVE918. Dispersions were estimated using estimateDisp with robust estimation. Differential gene expression analysis was conducted using edgeR (v4.6.3) [22] in R (4.5.0) and was assessed using the quasi-likelihood GLM framework implemented in glmQLFit and glmQLFTest. Pairwise contrasts were performed for OVE853 versus WT and OVE918 versus WT using makeContrasts. *p*-values were adjusted for multiple testing using the Benjamini–Hochberg method, and genes with an absolute log_2_ fold change ≥ 1.0 and a false discovery rate (FDR) ≤ 0.05 were designated as significantly differentially expressed. To assess global transcriptomic variation and sample relationships, principal component analysis (PCA) and multidimensional scaling (MDS) plots were generated using normalized expression data. No batch correction was applied because all samples were processed using the same library preparation protocol and sequencing workflow, and the PCA/MDS analyses did not identify any outlier samples. Top candidates were analyzed by using the iSyTE lens expression database as previously described [23,24,25] to examine their expression in the normal lens at different ages spanning embryonic through aging. A complete raw gene count matrix used for differential expression analysis is provided in Supplementary Table S2. Detailed RNA-seq preprocessing procedures are provided in Supplementary Data S1, and the complete R code used for downstream analyses is provided in Supplementary Data S2. The RNA-seq data generated in this study have been deposited in the NCBI Gene Expression Omnibus (GEO) under accession number GSE336537.

### 2.3. Gene Ontology (GO) Term Enrichment and Pathway Analysis

Gene Ontology (GO) enrichment analysis was performed using the clusterProfiler (v4.16.0) [26] package in R. This comprised independent analysis of GO Biological Process (BP), GO Molecular Function (MF), and GO Cellular Component (CC) to identify functional categories enriched among the differentially expressed genes (DEGs). Pathway-level analysis was also performed using curated gene sets from the Hallmark collection obtained from the msigdbr package (v25.1.1) [27,28] to identify coordinated biological pathways associated with the DEGs. For the comparison of DEGs in the *TGF-β1* overexpression and the post-cataract surgery (PCS) models [29,30,31,32], GO terms were identified using the “GOTERM_BP_DIRECT” annotation category using the bioinformatics tool DAVID [33,34]. The statistical significance of overlap was assessed using the hypergeometric distribution implemented in the R function phyper [35]. Directionality concordance was calculated as the proportion of overlapping genes exhibiting the same direction of differential expression in both datasets. For the 6 h PCS comparison, a *p*-value cut-off of <0.05 was used, while for the other stages, an FDR cut-off of <0.05 was used. A similar comparative analysis was performed using a published human capsular bag transcriptomic dataset (Cap24H vs. Cap0H) [36], and directionality concordance was assessed as described above.

### 2.4. Comprehensive Multi-Omics Platform for Biological InterpretatiOn (CompBio) Analysis

**Figure 1:**
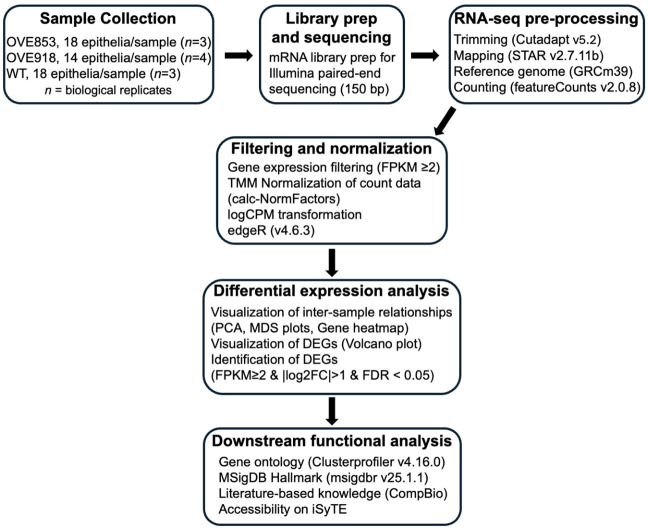
Overview of RNA-seq data processing and differential expression analysis pipeline for TGF-β1-overexpressing lens epithelium and control. Workflow illustrating the computational pipeline used for RNA-sequencing analysis to determine differentially expressed genes between WT and TGF-β1 overexpressed lens epithelia and the downstream analysis.

Enriched biological themes associated with the elevated and the reduced DEGs were identified using the Comprehensive Multi-omics Platform for Biological InterpretatiOn (CompBio), a web-based analytical tool developed at Washington University School of Medicine (GTAC@MGI, https://gtac-compbio-ex.wustl.edu (accessed on 10 April 2026)) [37]. CompBio analyzes gene lists to identify literature-derived biological concepts and groups the frequently co-occurring concepts into themes. Theme enrichment was evaluated using the normalized enrichment score (NEScore), and themes with an NEScore ≥ 1.3 and an associated *p*-value < 0.1 were considered significant. The identified significant themes were annotated to highlight key pathways and CompBio’s automated annotations were adopted where appropriate. An overview of the workflow for downstream data analysis is summarized in Figure 1.

## 3. Results

### 3.1. RNA-Seq Analysis of Isolated Lens Epithelia from the TGF-β1 Lens-Overexpressing Transgenic Mice

**Figure 2:**
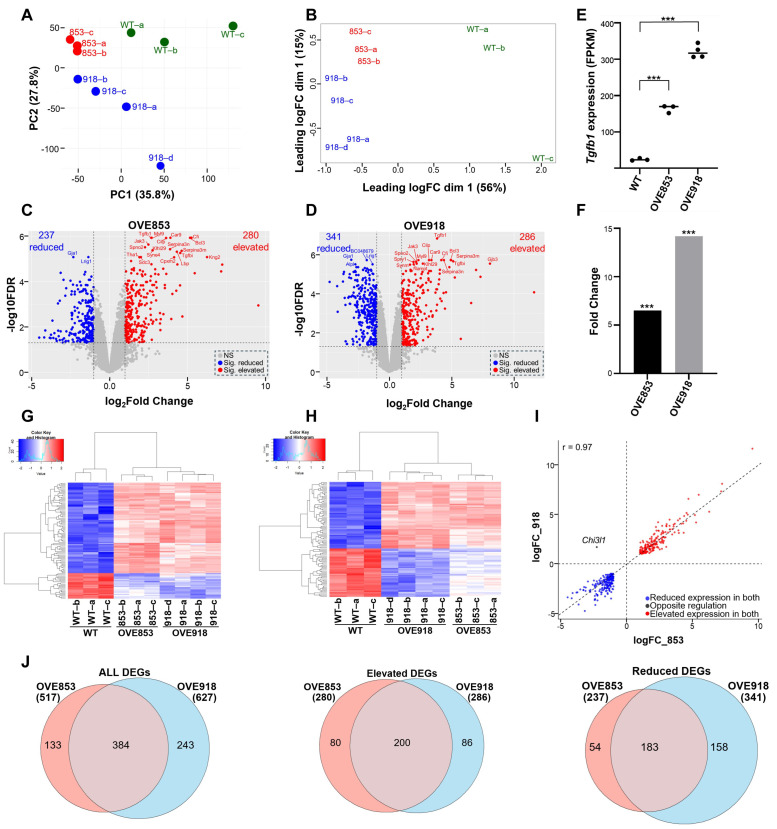
Transcriptomic profiling and differential expression analysis of *TGF-β1*-overexpressing lens epithelium and control. (**A**) Principal component analysis (PCA) of normalized gene expression data (logCPM) showing separation of control (WT) and TGF-β1-overexpressing samples. The two independent transgenic lines (OVE853 and OVE918) cluster distinctly from WT and from each other. (**B**) Multidimensional scaling (MDS) plot based on leading log-fold change distances demonstrates separation of samples by experimental group. (**C**,**D**) Volcano plots showing differential gene expression between (**C**) OVE853 versus WT and (**D**) OVE918 versus WT. Differentially expressed genes (DEGs) were defined using thresholds of false discovery rate (FDR) ≤ 0.05 and |log_2_ fold change| ≥ 1. Genes included in the analysis were filtered for expression ≥ 2 FPKM in at least half of all samples across WT and both transgenic lines. (**E**) Expression levels of *Tgfb1* (FPKM) across WT, OVE853, and OVE918 samples. Individual biological replicates are shown, with group means indicated. (**F**) Fold change in *Tgfb1* expression in OVE853 and OVE918 relative to WT. Statistical significance is indicated as *** (FDR < 0.001). Note: *Tgfb1* expression in (**E**,**F**) reflects both endogenous mouse *Tgfb1* and cross-mapped human *TGFB1* transgene-derived reads following alignment to mm39. (**G**,**H**) Heatmaps of the top significant 100 DEGs identified from comparisons of (**G**) OVE853 versus WT and (**H**) OVE918 versus WT. In each heatmap, expression patterns of the alternate transgenic line are also shown to enable cross-comparison. (**I**) Scatter plot comparing log_2_ fold changes in all shared genes between OVE853 and OVE918, demonstrating a strong positive correlation (r = 0.97). (**J**) Venn diagrams illustrating the overlap of DEGs between OVE853 and OVE918, including total DEGs, elevated genes, and reduced genes.

To gain insights into the transcriptomic changes induced by active *TGF-β1* overexpression in the mouse lens, we performed RNA-seq on lens epithelia isolated from the transgenic mouse lines OVE853 and OVE918 and wild-type (WT) controls at three weeks of age. Paired-end libraries (150 base pairs in length) were sequenced, and an average of 40.9 million reads were obtained per sample. Reads were aligned to the *Mus musculus* reference genome (GRCm39) using STAR (v2.7.11b) [19] with an average total mapping rate of 77.3%, including 74.1% uniquely mapped reads (Supplementary Table S1). First, we examined the data by performing principal component analysis (PCA) and multi-dimensional scaling (MDS). Both PCA and MDS showed that the datasets clustered largely based on sample type (Figure 2A,B). The RNA-seq analysis identified 10,566 genes to be expressed at ≥2.0 FPKM in at least six samples across the two transgenic lines and WT mice (Supplementary Tables S3 and S4). We applied stringent criteria to identify differentially expressed genes (DEGs) in comparisons between each of the transgenic lines and the control. The volcano plots for OVE853 vs. WT and OVE918 vs. WT highlight the DEGs that passed stringent criteria (log_2_ fold change ≥ 1.0, FDR ≤ 0.05, ≥2.0 FPKM) (Figure 2C,D). Importantly, *TGF-β1* was robustly elevated in both transgenic lines (Figure 2E,F). A total of 517 DEGs (280 elevated, 237 reduced) were identified in OVE853, while 627 DEGs (286 elevated, 341 reduced) were identified in OVE918 (Figure 2C,D; Supplementary Tables S5 and S6). Heatmap representations show the top 100 DEGs in the OVE853 vs. WT and the OVE918 vs. WT comparisons (Figure 2G,H). A total of 384 DEGs were commonly identified in both transgenic lines (Figure 2I,J, Supplementary Table S7). Among these candidates, 383 of the 384 DEGs shared the same trend in the context of differential expression (Figure 2I). Indeed, of the 384 DEGs, 200 were commonly elevated, while 183 were commonly reduced in both transgenic lines (Figure 2J). The magnitude of change in expression (in log_2_ fold change) of the majority of the genes correlated across both transgenic lines (*r* = 0.97) (Figure 2I), suggesting the robust nature of the commonly identified DEGs across OVE853 and OVE918. The top 20 commonly elevated genes and reduced genes in OVE853 and OVE918 are listed (Table 1).

### 3.2. Gene Ontology and Pathway Analysis of Common DEGs

**Figure 3:**
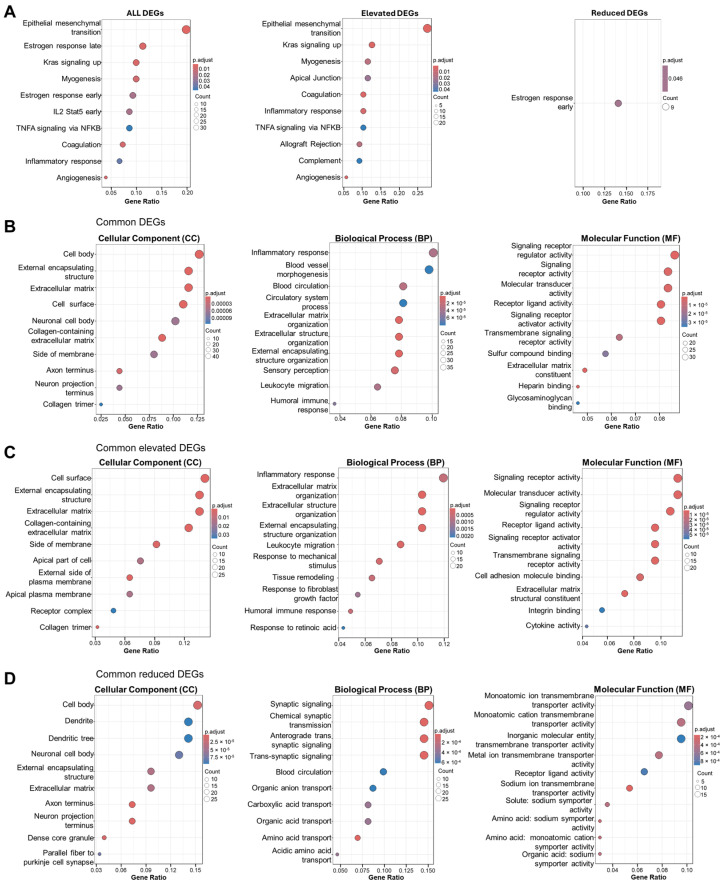
Pathway analysis of common differentially expressed genes in TGF-β1-overexpressing transgenic lens epithelia. (**A**) Hallmark gene set enrichment analysis using MSigDB (via msigdbr) was performed on all 384 common differentially expressed genes (DEGs), as well as on the 200 commonly elevated and 183 commonly reduced DEGs shared between OVE853 and OVE918. (**B**–**D**) Gene Ontology (GO) enrichment analysis of common DEGs across Cellular Component (CC), Biological Process (BP), and Molecular Function (MF) categories. (**B**) Enrichment results for all 384 common DEGs. (**C**) Enrichment results for the 200 commonly elevated DEGs. (**D**) Enrichment results for the 183 commonly reduced DEGs.

Pathway analysis and gene ontology (GO) analysis were next performed to gain insights into the common DEGs. Analysis of the 384 genes using the hallmark gene sets in the Molecular Signatures Database (MSigDB) [27,28] identified “Epithelial Mesenchymal Transition” among the top significantly enriched terms (Figure 3A), among other terms. Analysis of the 200 commonly elevated candidates identified “Inflammatory Response” in addition to “Epithelial Mesenchymal Transition” and other terms. Analysis of the 183 commonly reduced candidates identified only a single term, “Estrogen Response Early”. The complete MSigDB Hallmark enrichment results for the common, commonly elevated, and commonly reduced DEG sets are provided in Supplementary Tables S8–S10. Next, ClusterProfiler-based analysis of the 384 common DEGs identified the GO terms “Extracellular Matrix” (Cellular Component, CC), “Inflammatory Response” (Biological Process, BP), and “Signaling Receptor Activity” (Molecular Function, MF), among others (Figure 3B, Supplementary Table S11). Analysis of the 200 commonly elevated candidates identified “Extracellular Matrix” (CC), “Inflammatory Response”, “Response to Mechanical Stimulus” and “Response to Fibroblast Growth Factor” (BP), “Signaling Receptor Activity”, and “Cell Adhesion Molecule Binding” (MF) (Figure 3C, Supplementary Table S12). Analysis of the 183 reduced candidates identified “Neuronal Cell Body”, “Extracellular Matrix” (CC), “Synaptic Signaling”, “Organic Acid Transport” (BP) and Transporter Activity-related terms (MF) (Figure 3D, Supplementary Table S13). Together, these findings provide novel insights into the nature of the cell defects upon overexpression of active *TGF-β1* in the mouse lens epithelium.

### 3.3. TGF-β1 Overexpression Elevates EMT-Associated Genes in the Lens

**Figure 4:**
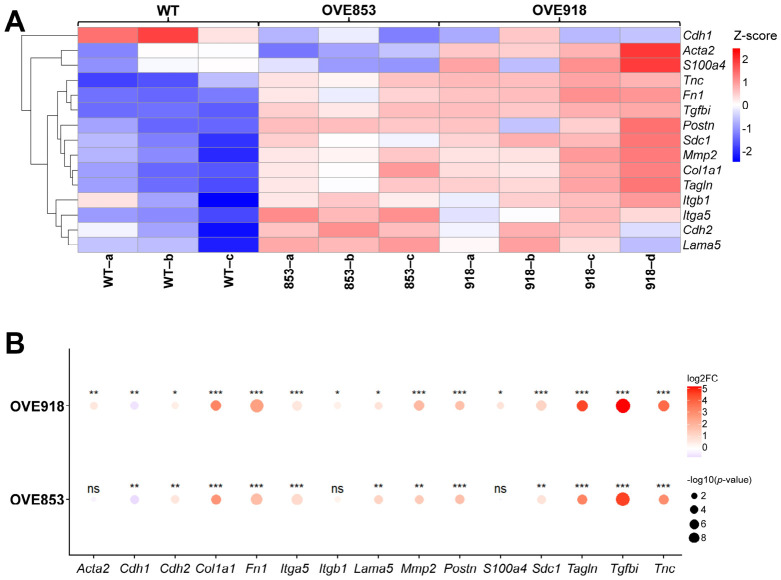
Expression patterns of epithelial-to-mesenchymal transition (EMT)-associated genes in TGF-β1-overexpressing transgenic lens epithelia. (**A**) Heatmap showing expression of EMT-associated genes across WT, OVE853, and OVE918 samples. Gene expression values (logCPM) were Z-score normalized across samples for each gene and visualized using a symmetric blue–white–red color scale centered at zero, where blue indicates relatively reduced expression, and red indicates relatively elevated expression. (**B**) Dot plot of the same EMT-associated genes comparing OVE853 and OVE918. Dot size reflects statistical significance, and color indicates the direction and magnitude of differential expression (log_2_ fold change). Statistical significance is indicated as follows: ns, not significant; * *p* < 0.05; ** *p* < 0.01; *** *p* < 0.001.

Because pathway analysis pointed to the elevation of EMT genes upon *TGF-β1* overexpression, we focused on candidates associated with EMT. The majority of 12 candidate genes (*Cdh2*, *Col1a1*, *Fn1*, *Itga5*, *Itgb1*, *Lama5*, *Mmp2*, *Postn*, *Sdc1*, *Tagln*, *Tgfbi*, and *Tmc*) associated with EMT—as per the literature—were found to be elevated in both transgenic lines (Figure 4A,B). Further, *Cdh1*, which is known to be downregulated with progressing EMT, was found to be reduced in both lines. Interestingly, *Acta2*, which encodes α-smooth muscle actin and is known to be elevated with EMT, was found to be elevated in OVE918 but not OVE853, as was *S100A4*.

### 3.4. TGF-β1 Overexpression Elevates a Subset of Inflammatory Response Genes in the Lens

**Figure 5:**
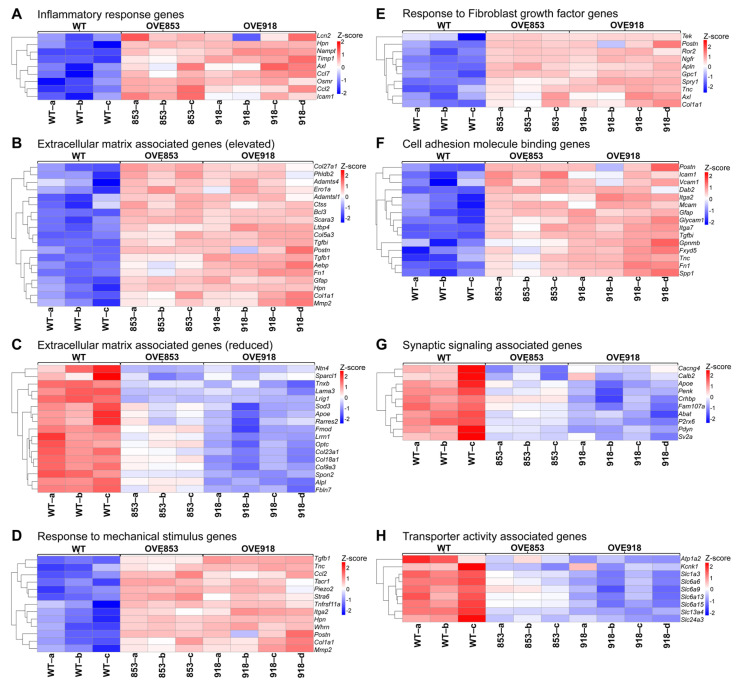
Functionally enriched categories among differentially expressed genes in TGF-β1-overexpressing transgenic lens epithelia. (**A**) Heatmap showing expression of inflammatory response genes across WT, OVE853, and OVE918 samples. (**B**,**C**) Heatmaps showing expression of extracellular matrix-associated genes derived from the enriched Gene Ontology (GO) term “extracellular matrix” among (**B**) commonly elevated and (**C**) commonly reduced differentially expressed genes (DEGs). (**D**–**F**) Heatmaps showing expression of genes associated with enriched GO terms among commonly elevated DEGs, including (**D**) response to mechanical stimulus, (**E**) response to Fibroblast Growth Factor, and (**F**) cell adhesion molecule binding. (**G**,**H**) Heatmaps showing expression of genes associated with enriched GO terms among commonly reduced DEGs, including (**G**) synaptic signaling and (**H**) transporter activity. For all panels, gene expression values (logCPM) were Z-score normalized across samples for each gene.

Pathway analysis showed that genes associated with the inflammatory response were elevated upon *TGF-β1* overexpression. To examine this in more detail, we focused on 9 candidates (*Axl*, *Ccl2*, *Ccl7*, *Hpn*, *Icam1*, *Lcn2*, *Nampt*, *Osmr*, and *Timp1*) associated with the inflammatory response as per the literature and found them to be elevated in both OVE853 and OVE918 (Figure 5A).

### 3.5. TGF-β1 Overexpression Dysregulates Extracellular Matrix Gene Expression in the Lens

Extracellular matrix (ECM) was identified as an enriched GO term by pathway analysis in both elevated and reduced DEGs. Among elevated DEGs, 19 genes (*Aebp1*, *Adamts4*, *Adamtsl1*, *Bcl3*, *Col1a1*, *Col27a1*, *Col5a3*, *Ctss*, *Ero1a*, *Fn1*, *Gfap*, *Hpn*, *Ltbp4*, *Mmp2*, *Phldb2*, *Postn*, *Scara3*, *Tgfb1*, and *Tgfbi*) were identified (Figure 5B). Among the reduced DEGs, 17 genes (*Alpl*, *Apoe*, *Col18a1*, *Col23a1*, *Col9a3*, *Fbln7*, *Fmod*, *Lama3*, *Lrig1*, *Lrrn1*, *Ntn4*, *Optc*, *Rarres2*, *Sod3*, *Sparcl1*, *Spon2*, and *Tnxb*) were identified (Figure 5C). It should be noted that a majority of the candidates with reduced expression and GO-enriched ECM terms are known to be involved in ECM organization and are not associated with fibrosis (e.g., *Col18a1*, *Lama3*), unlike those identified as elevated and GO-enriched ECM. These findings suggest that *TGF-β1* overexpression may result in a complex interplay of mis-expressed genes associated with ECM remodeling in the lens.

### 3.6. TGF-β1 Overexpression in the Lens Elevates Several Genes Linked to Mechano-Sensation, FGF Signaling and Cell Adhesion

*TGF-β1* overexpression leads to elevated expression of several genes that are associated with the GO term for response to mechanical stimulus. These include *Ccl2*, *Col1a1*, *Hpn*, *Itga2*, *Mmp2*, *Piezo2*, *Postn*, *Stra6*, *Tacr1*, *Tgfb1*, *Tnc*, *Tnfrsf11a* and *Whrn* (Figure 5D). Several genes that are linked to the GO for Fibroblast Growth Factor (FGF) signaling are found to be elevated upon *TGF-β1* overexpression in the lens. These are *Apln*, *Axl*, *Col1a1*, *Gpc1*, *Ngfr*, *Postn*, *Ror2*, *Spry1*, *Tek*, and *Tnc* (Figure 5E). Genes linked to the GO term for cell adhesion that were elevated in *TGF-β1* overexpression lines were *Dab2*, *Fn1*, *Fxyd5*, *Gfap*, *Glycam1*, *Gpnmb*, *Icam1*, *Itga2*, *Itga7*, *Mcam*, *Postn*, *Spp1*, *Tgfbi, Tnc* and *Vcam1* (Figure 5F).

### 3.7. TGF-β1 Overexpression in the Lens Reduces Genes Associated with Synaptic Signaling and Transporter Activity

Overexpression of *TGF-β1* led to a reduction in a cohort of genes linked to GO terms of synaptic signaling and transporter activity. Interestingly, most of these genes were also found to be elevated in the normal lens epithelium. Genes associated with synaptic signaling that were reduced upon *TGF-β1* overexpression in the lens were *Abat*, *Apoe*, *Cacng4*, *Calb2*, *Crhbp*, *Fam107a*, *P2rx6*, *Pdyn*, *Penk*, *Sv2a*, etc., while those linked to transporter activity were *Atp1a2*, *Kcnk1*, *Slc1a3*, *Slc6a6*, *Slc6a9*, *Slc6a13*, *Slc6a15*, *Slc13a4*, and *Slc24a3* (Figure 5G,H). The reduced expression of these genes may likely contribute to the lens defects observed in the transgenic mouse lines OVE853 and OVE918.

### 3.8. CompBio Analysis of Genes Differentially Expressed upon TGF-β1 Overexpression

Next, we sought to apply an ontology-independent strategy—distinct from the above GO/pathway analyses—to examine the *TGF-β1* overexpression common DEGs in OVE853 and OVE918 mouse lens epithelia. Therefore, we used CompBio (Comprehensive Multi-omics Platform for Biological InterpretatiOn) [37] that analyzes de novo a given list of genes (e.g., elevated or reduced or differentially expressed) using publicly available literature resources encompassing >30 million PubMed abstracts and >3 million full-text articles to build biologically relevant themes. Distinct themes were identified when elevated genes and reduced genes were analyzed separately by CompBio.

**Figure 6:**
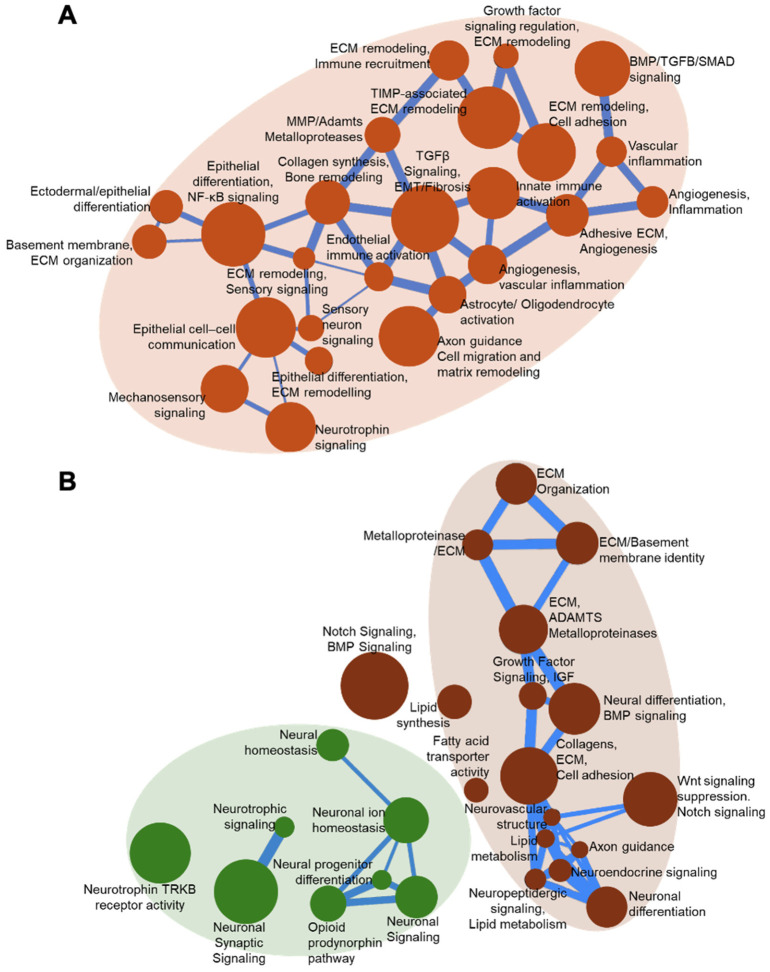
CompBio analysis identifies enriched biological themes among common differentially expressed genes in TGF-β1-overexpressing transgenic lens epithelia. (**A**,**B**) CompBio-based analysis of biological themes enriched among (**A**) commonly elevated and (**B**) commonly reduced genes shared between TGF-β1-overexpressing lines. Significantly enriched themes were defined by *p*-value ≤ 0.1 and normalized enrichment score (NEScore) ≥ 1.3.

Analysis of the significantly elevated genes by CompBio uncovered “TGFβ signaling, EMT, Fibrosis” as the topmost enriched theme (Figure 6A). While this is not surprising, it shows the efficacy of CompBio. Other top themes identified include “TIMP (tissue inhibitor of metalloproteinases)-associated ECM remodeling”, “Epithelial cell communication”, and “Axon guidance, Cell migration and Matrix remodeling”. In CompBio-based analysis of the significantly reduced genes, the themes identified include “Notch signaling, BMP signaling”, “Wnt signaling suppression, Notch signaling”, “Growth factor signaling”, “Lipid metabolism”, “Neuronal ion homeostasis” and “Neuronal synaptic signaling” (Figure 6B). Thus, CompBio analysis provides additional biological insights into the pathways that are altered upon *TGF-β1* overexpression in OVE853 and OVE918 lens epithelia.

### 3.9. Comparison of Temporal Transcriptomes of TGF-β1 Overexpression DEGs with a Mouse Cataract Surgery Model and a Human Capsular Bag Model of Posterior Capsular Opacification

**Figure 7:**
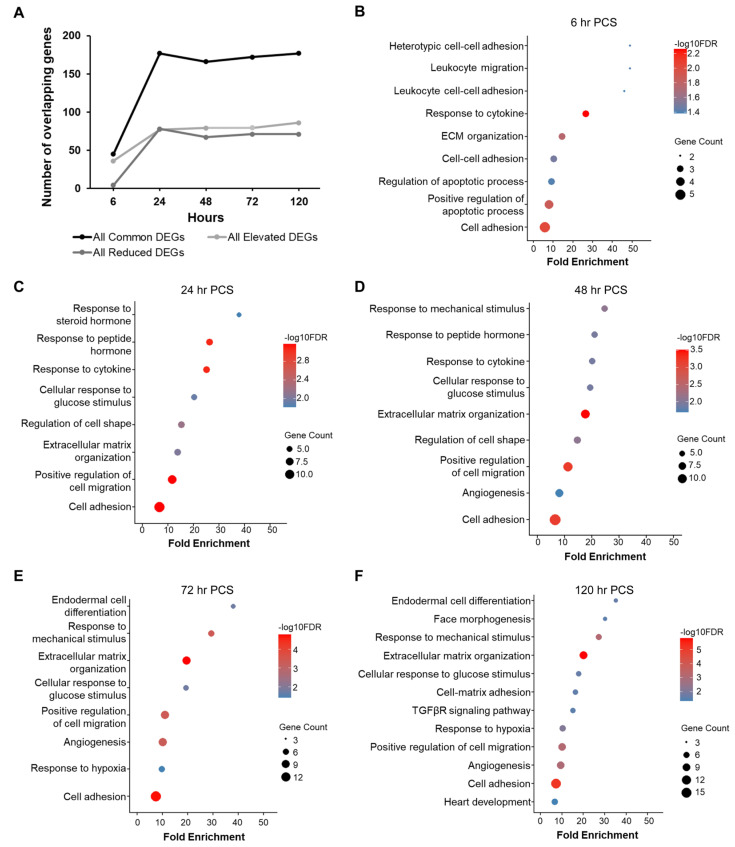
Comparison of TGF-β1-overexpression-associated differentially expressed genes with gene expression changes across different stages in a mouse post-cataract surgery model. (**A**) The graph shows the number of differentially expressed genes (DEGs) shared between TGF-β1 overexpression (*n* = 384 common DEGs) and a published post-cataract surgery model across multiple time points. A total of 45 DEGs were shared at 6 h, and 177, 166, 172, and 177 DEGs were shared at 24, 48, 72, and 120 h, respectively. Shared DEGs were further categorized as commonly elevated or reduced at each time point. Gene ontology (GO) Biological Process term enrichment in common elevated DEGs between TGF-β1 overexpression and the post-cataract surgery (PCS) model at (**B**) 6 h PCS, (**C**) 24 h PCS, (**D**) 48 h PCS, (**E**) 72 h PCS and (**F**) 120 h PCS. Log2FDR and gene count are given for each time point.

*TGF-β1* upregulation is associated with posterior capsule opacification (PCO). Previous studies have generated transcriptome data on an established mouse cataract surgery model for PCO [29,30,31,32]. To gain insights into the shared pathways, we performed a comparative analysis with transcriptome data collected on different stages post-cataract surgery in the mouse cataract surgery model. Comparison of our *TGF-β1* overexpression DEGs (*n* = 384) with those in the 6 h post-cataract surgery (PCS) model identified 45 common DEGs (Figure 7A, Supplementary Table S14). At 24 h, 48 h, 72 h and 120 h post cataract surgery, 177, 166, 172 and 177 DEGs were commonly identified, respectively, between the cataract surgery model and the *TGF-β1* overexpression lens epithelia. For 6 h, 36 were commonly elevated (and 4 commonly reduced), for 24 h, 77 were commonly elevated (and 78 commonly reduced), for 48 h, 79 were commonly elevated (and 67 commonly reduced), for 72 h, 79 were commonly elevated (and 71 commonly reduced), and for 120 h, 86 were commonly elevated (and 71 commonly reduced) (Figure 7A, Supplementary Table S14). Hypergeometric testing demonstrated that the overlap between the TGF-β1-overexpression DEGs and PCS datasets was significantly greater than expected by chance across all PCS time points (Supplementary Table S15). Directionality concordance analysis further showed that 87–89% of overlapping genes exhibited concordant regulation between the datasets (Supplementary Table S16). Next, we performed GO Biological Process term enrichment analysis in DEGs shared between TGF-β1 overexpression and the PCS model at 6 h (Figure 7B), 24 h (Figure 7C), 48 h (Figure 7D), 72 h (Figure 7E) and 120 h (Figure 7F). At 6 h PCS, “Response to cytokine” and “Cell adhesion” were identified among the enriched terms. No GO terms associated with the 6 h overlap gene set were significant after multiple-testing correction. However, to avoid overlooking candidates of potential biological significance, for just the 6 h set, only nominally significant terms (*p* < 0.05) are reported. For the later stages, “Positive regulation of cell migration”, “Extracellular matrix organization”, “Cell adhesion”, “Response to mechanical stimulus”, and “TGFβR signaling pathway” were identified among the enriched terms. To assess the relevance of these findings to human lens pathology, we compared the 384 common TGF-β1-overexpression DEGs with a published human capsular bag model of posterior capsular opacification for which the transcriptomic dataset is available (0 h (Cap0H), 24 Hr (Cap24H) after fiber removal) [36]. This analysis identified 116 overlapping DEGs (30.5%), of which 83 (70.9%) exhibited concordant regulation between the datasets, including 42 genes elevated and 41 genes reduced in both datasets (Supplementary Table S17).

### 3.10. Identification of ASC-Associated ER Stress Genes and Novel Candidates in TGF-β1 Overexpression Transcriptome Data

**Figure 8:**
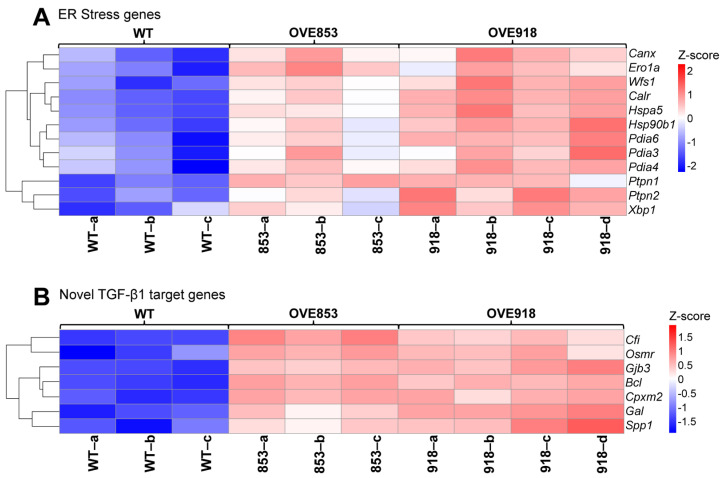
Expression of endoplasmic reticulum (ER) stress-associated genes in TGF-β1-overexpressing transgenic lens epithelia. (**A**) Heatmap showing expression of ER stress-associated genes, derived from pathways reported to be altered in human lenses with anterior subcapsular cataract (ASC), across WT, OVE853, and OVE918 samples. (**B**) Heatmap showing expression of promising candidate genes elevated in TGF-β1-overexpressing transgenic lens epithelia across WT, OVE853 and OVE918 samples. Gene expression values (log_2_CPM) were Z-score normalized across samples for each gene.

*TGF-β1* overexpression transcriptome data analysis offers an opportunity to identify new directions downstream of the pathway with relevance to ASC. Toward this goal, we first compared the *TGF-β1* overexpression common DEGs to genes that showed altered expression in a recent study of human ASC [38]. This analysis identified ER stress genes to be elevated in both *TGF-β1* transgenic lens epithelia datasets (Figure 8). To investigate this in more detail, we examined other ER stress genes beyond the ones that were identified in the human dataset and identified a cohort of significantly elevated genes in the *TGF-β1* overexpression mouse models. The ER stress genes elevated are: *Calr*, *Canx*, *Ero1a*, *Hsp90b1*, *Hspa5*, *Pdia3*, *Pdia4*, *Pdia6*, *Ptpn1*, *Ptpn2*, *Wfs1*, and *Xbp1* (Figure 8A). Next, we focused on identifying genes other than those related to ER stress, but that have not been previously linked to *TGF-β1*. Examination of our data prioritized several novel genes that are significantly elevated upon *TGF-β1* overexpression. These are: *Bcl3*, *Cfi*, *Cpxm2*, *Gal*, *Gjb3*, *Osmr*, and *Spp1* (Figure 8B). These genes exhibit low expression in normal lens epithelium in iSyTE and are found to be elevated upon *TGF-β1* overexpression.

### 3.11. Accessing the TGF-β1 Overexpression Transcriptome Data in iSyTE

**Figure 9:**
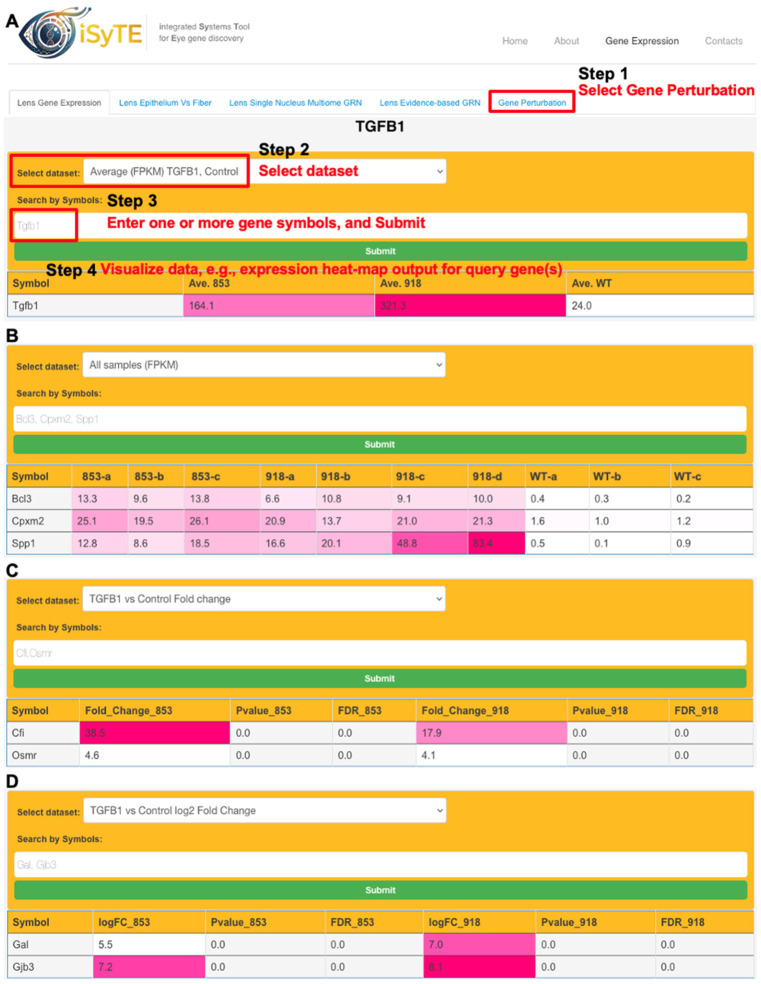
Access and user-friendly visualization of TGF-β1 overexpression and control lens epithelium transcriptome data in iSyTE. (**A**) The TGF-β1 overexpression lens epithelium transcriptome data is made freely accessible in a new user-friendly web portal on the iSyTE webpage at http://research.bioinformatics.udel.edu/iSyTE (accessed on 17 April 2026). This provides effective visualization of one or more genes in the lens epithelium of the two TGF-β1 overexpression transgenic lines, OVE853 and OVE918, by following Steps 1 through 4. The example given is of Tgfb1 that shows average high expression in fragments per kilobase of transcript per million mapped reads (FPKM) in both OVE853 and OVE918 compared to wild-type (WT) lens epithelium. (**B**) Selection of dataset “All samples (FPKM)”, (**C**) or “TGFB1 vs. Control Fold Change”, (**D**) or “TGFB1 vs. Control log2 Fold Change”, shows visualization of candidate genes in each of the replicates of OVE853, OVE918, and control, or for fold change or log2 fold change differential gene expression. Color intensity reflects the magnitude of the displayed values (expression, fold change, or log2 fold change), with white/light pink indicating relatively lower values and progressively darker pink indicating relatively higher values.

We sought to make the *TGF-β1* overexpression lens epithelia transcriptome data freely accessible. For this, we developed a new user-friendly web portal on the iSyTE database [23] that can be navigated at http://research.bioinformatics.udel.edu/iSyTE (accessed on 17 April 2026). This allows effective visualization of the transcriptome data, including genes that are expressed in the *TGF-β1* overexpressing lens epithelia. Expression of one (or more) candidates can be visualized through these new features at iSyTE. The database resource is built such that one can navigate to the iSyTE webpage, select “Gene Expression”, “Gene Perturbation”, select “TGFB1 Over-Exp”, select dataset “Average (FPKM) TGFB1, Control” (e.g., for average FPKM values for individual genes detected in transgenic lines OVE853, OVE918 and control). For example, elevated *Tgfb1* expression in the transgenic lines compared to the control can be visualized (Figure 9A). Other features can also be selected for selective visualization. For example, select dataset “All samples (FPKM)” for visualization of individual genes in each of the replicates of OVE853, OVE918, and control, or “TGFB1 vs. Control Fold Change” or “TGFB1 vs. Control log2 Fold Change”, for fold change or log2 fold change differential gene expression. Examples are shown for differentially expressed genes with elevated or reduced expression in the transgenic lines compared to the control (Figure 9B–D). Together, these data demonstrate that the new web-portal-enabled data visualization of the RNA-seq data in iSyTE allows effective examination of differential gene expression in normal and *TGF-β1* overexpression lens epithelia.

## 4. Discussion

Here, we performed a high-throughput RNA-seq analysis of isolated lens epithelia from well-established transgenic mouse lines OVE853 and OVE918 overexpressing human self-activating TGF-β1; models for human ASC. Although independently derived, both transgenic lines exhibited 384 common differentially expressed genes, suggesting a high-level of overlap in their transcriptomes. Importantly, these data show *TGF-β1* is indeed robustly overexpressed in both lines, as are many of their downstream targets. Furthermore, analysis of the 384 common dysregulated genes confirms many of the pathways and genes that are expected to be perturbed in these lines, based on their biochemical, cellular, and phenotypic characterization from previous studies. Indeed, genes aberrantly expressed in both lines include those associated with EMT and ECM remodeling, typical of ASC and fibrotic forms of cataract. Our past immunolabeling studies independently validate important candidate genes identified in our current analysis, for example, elevated levels of Collagen type I (COL1A1, elevated 3.2-fold), Tenascin C (TNC, elevated 3.8-fold), and Fibronectin (FN1, elevated 2.5-fold) [4], to name a few.

From the outset, it should be noted that TGF-β1 overexpression in the transgenic mouse lens primarily leads to ASC plaque development in the central lens epithelia, similar to that found in humans. In these lines, within one week postnatally, we see disruption of the central lens epithelial sheet, progressively maturing into fibrotic plaques over three weeks [4]. As this analysis was performed on the complete epithelium, rather than the isolated region of the ASC plaque, the differential gene expression measurements reported may be effectively “diluted” by “normal” or “non-ASC” cell epithelia. In short, the fold-changes observed for the DEGs in the TGF-β1 overexpression lens epithelia, while highly relevant, may be an underestimate of the extent of gene mis-expression specific to the ASC plaque region. Moreover, while both transgenic lines exhibited highly similar transcriptional profiles, any differences in the magnitude of gene expression may simply reflect a more pronounced molecular response.

These new transcriptome analyses give novel insights into the impact of active *TGF-β1* in the lens epithelium. For example, *TGF-β1* overexpression led to a reduction in genes linked to the lens epithelium, such as the E-cadherin gene *Cdh1*, the connexin gene *Gja1,* and the glutathione peroxidase gene *Gpx3*. Notably, *GJA1* mutations are associated with human syndromic cataract [39,40]. Further, *TGF-β1* overexpression impacted multiple signaling pathways in the lens epithelia. For example, *Bmp4* and *Bmp7*, which exhibit elevated expression in the normal lens epithelium compared to normal fibers [25], were found to be significantly reduced in *TGF-β1* overexpression transgenic lines, suggesting perturbation of Bmp signaling. Interestingly, Bmp4 downstream targets, *Id4*, and the homeodomain transcription factor *Msx1* (which also exhibits elevated expression in normal lens epithelium compared to fibers), were also reduced. These transcriptome changes, along with the normally epithelium-elevated genes discussed above, are consistent with the expected loss of lens epithelial character upon *TGF-β1* overexpression. Further, BMP4 and BMP7 have been shown to inhibit EMT in lens explants [41] and in an ASC model by reducing components of the Notch pathway [42]. Interestingly, in the present study, overexpression of *TGF-β1* had an opposite effect on the two different Notch pathway ligands, *Jag1* and *Dll1*; the former was elevated while the latter was reduced. Moreover, the Notch downstream targets, *Hes1* and *Hes5*, were found to be reduced, as were the Notch pathway modulators *Dtx1* (Notch negative regulator) and *Dtx4* (Notch positive regulator), suggesting that *TGF-β1* overexpression led to dysregulation of multiple components associated with the Notch pathway, but in a complex manner.

Previous studies have reported a significant increase in ER stress-associated gene expression in human ASC lenses [38]. Consistent with these observations, our analysis detected elevated expression of ER stress-associated genes in TGF-β1-overexpressing mouse lens epithelia. This finding suggests that *TGF-β1* signaling may contribute to ASC pathogenesis through the activation of ER stress pathway genes and additionally demonstrates the utility of these transgenic mouse lines for modeling human ASC. Together, these data uncover key themes associated with elevated or reduced genes upon *TGF-β1* overexpression in OVE853 and OVE918 mouse lenses.

TGF-β signaling has been implicated in the pathogenesis of PCO, a secondary cataract that arises following lens surgery and is characterized by EMT and fibrotic remodeling of lens epithelial cells and capsule [43,44,45]. Importantly, ASC, as observed in the *TGF-β1* overexpression transgenic models, shares key pathological features with PCO, including aberrant ECM deposition, enhanced cell migration, and fibrotic plaque formation. Given these shared fibrotic characteristics and the central role of TGFβ signaling in both contexts, we highlighted the extent to which regulatory programs induced by *TGF-β1* overexpression in the mouse lens epithelia overlap with those activated in the mouse cataract surgery (lens injury) model developed for studying PCO [46]. When we compared our *TGF-β1* overexpression RNA-seq data with the previously generated RNA-seq data on different time points post-cataract surgery, at early stages of PCS, there was a ~12% overlap between the DEGs, including candidate genes enriched for acute inflammatory processes such as response to cytokine and cell adhesion. Given that no GO terms associated with the 6 h overlap remained significant after multiple-testing correction, the 6 h enrichment analysis is considered exploratory. At later PCS stages the degree of overlap between the two models increased, and remained relatively stable (~43–46% overlap, consistently enriched for pathways associated with cellular activation and tissue remodeling, including response to cytokine, cell adhesion, positive regulation of cell migration, ECM organization, regulation of cell shape, response to mechanical stimulus and angiogenesis-related processes. In the PCS model, genes associated with the inflammatory response are elevated early after surgery, with *TGF-β1* levels similar to that at 0 h PCS but get progressively elevated at later stages. In the *TGF-β1* overexpression transgenic lines as well, we find cytokine-response pathway to be elevated with significant FDR. These comparative data analyses suggest that TGF-β1 overexpression is sufficient to induce a subset of inflammation-associated transcriptional programs, independent of physical injury associated with PCS, and may contribute to transcriptional programs associated with fibrotic responses. These findings are further supported by comparison with a published human capsular bag model of posterior capsular opacification for which transcriptomic data was available. This analysis identified substantial overlap and concordant regulation of TGF-β1-overexpression DEGs, indicating that these models share components of the transcriptional response associated with human lens injury. However, the PCS models represent an acute injury response, whereas human PCO typically develops over a substantially longer time frame following cataract surgery. Thus, while these transcriptomic similarities indicate shared wound-healing, inflammation-associated, and fibrotic responses, they do not establish a common pathogenesis or identify TGF-β1 as the initiating factor in human PCO.

Further, these data identify several new candidate genes associated with TGF-β1 overexpression that warrant further investigation in the context of lens pathology. For example, we find the known TGF-β1 downstream gene, *Tgfbi* (Transforming Growth Factor-beta-induced), which encodes a collagen-binding RGD-domain protein, is highly elevated in *TGF-β1* overexpressing lens epithelia. *Tgfbi*, previously linked with corneal dystrophy [47], has been implicated in cell adhesion remodeling and fibrosis [48] and is therefore a promising candidate for further examination in ASC.

Our examination using two independent pathway analysis tools, GO representation and CompBio, reveals an enrichment of biological themes related to inflammation in the elevated DEGs. This offers support to a model in which TGF-β1 overexpression in lens epithelia likely contributes to an inflammatory response that, in a feedback loop, may contribute to fibrosis. Additionally, both analyses independently identify “Mechanosensory signaling” (CompBio) and “Response to mechanical stimulus” (GO) among the elevated DEGs. There are four common genes—*Piezo2*, *Tacr1*, *Tnc*, and *Whrn*—that contribute to the enrichment of these themes in both approaches. Piezo2 is a mechanically activated ion channel that converts mechanical stimulus into electrical signals to provide physical sensory stimulation [49]. Tacr1 encodes a G-protein coupled receptor (tachykinin receptor 1) [50], Tnc encodes an ECM protein with EGF-like and fibronectin type-III domains (tenascin C) [51], and Whrn encodes a PDZ-domain protein (whirlin) [52]—all being implicated in various roles in mechano-sensing. Interestingly, examination of another gene implicated in mechano-sensing and lens pathology, *Piezo1*, in the LIRTS Viewer, a web-based resource for PCS RNA-seq data visualization [32], indicates that it remains elevated in PCS from 6 h. In contrast, the LIRTS server shows that *Piezo2* is robustly elevated at 72 h and 120 h PCS in the mouse model, after the elevation of active TGF-β1 at 48 h [53]. It is plausible that *Piezo2* elevates after a threshold of active TGF-β1 levels is achieved. This agrees with our data that shows *Piezo2* to be significantly elevated upon overexpression of active TGF-β1. Thus, our work identifies *Piezo2* to be a candidate gene associated with TGF-β1 overexpression, suggesting TGF-β1′s role in response to mechanical stimuli. The mechanical stimuli may reflect changes in ECM and cellular properties arising from TGF-β1 overexpression. The enrichment of these biological themes in response to TGF-β1 overexpression in lens epithelia suggests an overlap with the post-cataract surgery response program in the mouse model.

Among the reduced DEGs, both GO and CompBio analysis also identified terms related to lipid metabolism. TGF-β1 overexpression in lens epithelia reduces expression of *Acsl3* (acyl-CoA synthetase long chain family member 3), *Apoe* (apolipoprotein E), *Hmgcs2* (3-hydroxy-3-methylglutaryl-CoA synthase 2), *Insig1* (insulin-induced gene 1), *Mgll* (monoglyceride lipase), *Pxmp2* (peroxisomal membrane protein 2), and *Scd1* (stearoyl-Coenzyme A desaturase 1). These genes are involved in different aspects of lipid metabolism, including regulation of fatty acid activation, transport, storage, and biosynthesis and several candidates (e.g., *Acsl3*, *Apoe*, and *Scd1*) have been implicated in other conditions, e.g., tumor progression and neurodegenerative disorders. Consistent with our findings, previous lipidomics studies have shown that TGF-β signaling broadly reduces lipid metabolites (~100 out of 130 metabolites reduced) in lens epithelial cells [38], supported by recent lipidomic profiling of transcriptomic datasets in lens EMT [54]. Our data, along with these studies, offer support to the hypothesis that TGF-β1 overexpression disrupts lipid homeostasis in the lens epithelia, which may contribute, perhaps via ER stress, to the pathological changes in the lens.

This transcriptome analysis also identifies several candidate genes associated with TGF-β1 overexpression in the lens, which may represent targets for future investigations. Indeed, several genes (e.g., *Bcl3*, *Cfi*, *Cpxm2*, *Gal*, *Gjb3*, *Osmr*, and *Spp1*) were identified as candidate downstream components of the TGF-β1-associated transcriptional response, although further studies are required to establish direct regulation. For example, Bcl3 (BCL3 transcription coactivator; encodes an ankyrin repeat containing protein functioning as transcriptional co-activator with NF-kappa), Cfi (complement factor I encodes a serine protease involved in complement cascade), *Cpxm2* (carboxypeptidase X, M14 family member 2, encodes a predicted extracellular protein with proteolytic activity), *Gal* (galanin and GMAP prepropeptide encodes a precursor for peptides with diverse functions including osmotic regulation and potentially linked to innate immunity), *Gjb3* (gap junction protein beta 3 encodes a connexin/gap junction protein intercellular channel), *Osmr* (oncostatin M receptor encodes a type I cytokine receptor, heterodimerizes with interleukin 6 signal transducer and interleukin 31 receptor A to form their respective receptors) and *Spp1* (secreted phosphoprotein 1 encodes a secreted protein that binds hydroxyapatite and can function as a cytokine that upregulates interferon-gamma and interleukin-12) were all found to be elevated upon TGF-β1-overexpression. Interestingly, several neuronal signaling-associated genes were also misregulated in both transgenic lines. Although the lens is not a neuronal tissue, aspects of neuronal gene expression have been reported in the normal lens [55,56]. Furthermore, there is growing evidence that the lens retains regulatory mechanisms for controlling neuronal gene expression, misexpression of which is correlated with lens pathology [57,58]. This provides added evidence that TGF-β1 overexpression induces widespread transcriptomic reprogramming in lens epithelial cells. Further, a recent transcriptome study on TGF-β2-treated primary lens epithelial cells [59] identified ECM, positive regulation of cell migration, cytokine activity, cell–cell junction, etc., among the GO categories for the DEGs, similar to the GOs identified in the DEGs in our analysis. Future studies will independently validate key novel candidate genes identified in this analysis using qRT-PCR and protein-level analyses.

As mentioned earlier, given that we did not perform our analysis on the isolated regions of ASC plaques, but on whole epithelia (effectively underestimating the extent of gene mis-expression specific to the ASC plaque), in future studies, this can be addressed by performing laser capture microdissection (LCM) of the ASC plaque epithelial region and comparing its transcriptome with the non-ASC epithelial region. This can also be addressed by performing single-cell or single-nucleus RNA-seq or multiomics (combined RNA-seq and ATAC-seq), which would allow cell-population-specific analysis [60]. A spatial transcriptomics approach could also be applied to address these limitations. Notably, while the transgenic mouse models overexpress active TGF-β1 in lens cells from embryonic stages, disruption of the central lens epithelia leading to the formation of ASC plaques commences postnatally. Because these plaques are isolated, it seems that *TGF-β1* overexpression does not drive the lens epithelium en masse into an ASC fate but does so in a localized manner. The properties that make the central epithelial cells more susceptible to TGF-β1-induced ASC in situ, compared to others, are a subject for future investigation. Because the TGF-β1 transgene is overexpressed in both epithelial and fiber cells in these models, future studies examining the effect of TGF-β1 overexpression on lens fiber cell transcriptomes will help determine whether these compartments exhibit any shared or distinct molecular responses. Although these findings provide insight into TGF-β1-induced ASC pathology, their clinical relevance requires validation in ASC with further studies.

## 5. Conclusions

**Figure 10:**
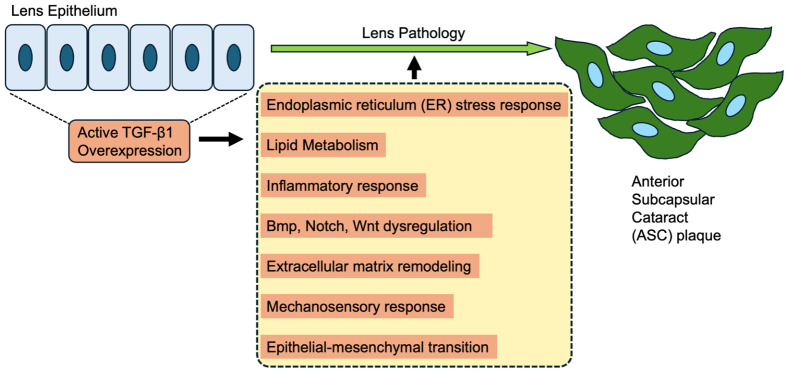
Schematic summary of biological pathways associated with TGF-β1 overexpression in the anterior subcapsular cataract (ASC) mouse model. Active TGF-β1 overexpression in the mouse lens epithelium is associated with altered expression of genes involved in multiple biological pathways, including endoplasmic reticulum (ER) stress response, lipid metabolism, inflammatory response, Bmp, Notch and Wnt signaling, extracellular matrix remodeling, mechanosensory response, and epithelial-to-mesenchymal transition. These transcriptomic changes are associated with the ASC phenotype observed at 3 weeks of age in the transgenic models. Note that these transgenic models phenotype features of human ASC, e.g., dysregulation of genes in reticulum (ER) stress response, and lipid metabolism.

In summary, this work presents the first detailed transcriptome analyses of the impact of overexpression of active TGF-β1 in the lens epithelium. Based on this data, our model demonstrates that distinct pathways associated with ER stress and lipid metabolism are mis-expressed in response to active TGF-β1 overexpression (Figure 10). Along with misregulation of other pathways, such as inflammatory response and signaling pathways (Bmp, Notch, and Wnt), this likely feeds into ECM remodeling, mechanosensory response, and EMT, culminating in anterior subcapsular cataract. Our work aligns with previous findings that link ER stress and lipid metabolism misregulation with human anterior subcapsular cataract. This work also uncovers the genes and the pathways shared by TGF-β1 overexpression and post-cataract surgery response of lens epithelial cells, also identifying several candidate genes associated with TGF-β1 in epithelial cell biology for future analyses. Taken together, these data provide novel molecular insights into the transcriptomic changes associated with anterior subcapsular cataract.

## Figures and Tables

**Table 1:** Top 20 common elevated DEGs and 20 common reduced DEGs between OVE853 and OVE918 lens samples with their associated log2FC (fold change) values in both lines.

Gene	Log2FC_853	Log2FC_918	Gene	Log2FC_853	Log2FC_918
*Opalin*	9.55	11.61	*Slc6a13*	−3.17	−4.77
*Gjb3*	7.24	8.07	*Calb2*	−4.49	−3.39
*Zim1*	7.17	7.3	*Aldh3a1*	−3.64	−4.11
*Gal*	5.47	6.95	*Sncg*	−4.18	−3.48
*Kng2*	6.26	5.25	*Cartpt*	−4.10	−2.94
*Spp1*	4.68	6.55	*Stmn3*	−3.82	−2.91
*Bcl3*	5.17	4.92	*Gng13*	−3.74	−2.88
*Tgfbi*	4.55	5.12	*Best2*	−2.39	−4.16
*Serpina3m*	4.63	4.96	*Cplx3*	−3.52	−2.58
*Cfi*	5.27	4.16	*Atp1a2*	−2.43	−3.66
*Gjb2*	4.36	4.81	*Wfdc1*	−2.41	−3.62
*H19*	4.53	4.34	*Penk*	−2.40	−3.56
*Gm40376*	4.04	4.54	*Mt1*	−3.29	−2.66
*Gm266*	4.3	4.07	*Rlbp1*	−3.29	−2.65
*Serpina3n*	3.89	4.37	*Fam107a*	−2.23	−3.70
*Cpxm2*	4.15	4.05	*Sgk1*	−3.12	−2.74
*Tagln*	3.25	4.46	*Fat4*	−2.42	−3.23
*Serpina3g*	3.65	4.03	*Scg2*	−3.24	−2.36
*Col5a3*	3.56	3.96	*Lama3*	−2.76	−2.83
*Lbp*	4.33	3.14	*Thrsp*	−2.83	−2.72

## Data Availability

The original data presented in the study are openly available in the NCBI Gene Expression Omnibus (GEO) at GEO Accession viewer (accession number GSE336537).

## Supplementary Materials

The following supporting information can be downloaded at https://www.mdpi.com/article/10.3390/cells15141263/s1. Table S1: RNA-seq QC metrics; Table S2: Raw gene count metrics; Table S3: List of all genes expressed in OVE853; Table S4: List of all genes expressed in OVE918; Table S5: List of significantly differentially expressed genes in OVE853; Table S6: List of significantly differentially expressed genes in OVE918; Table S7: List of common 384 DEGs and their expression in FPKM across all samples; Table S8: MSiGDB Hallmark analysis for all 384 common DEGs; Table S9: MSiGDB Hallmark analysis for 200 common elevated DEGs; Table S10: MSiGDB Hallmark analysis for 183 common reduced DEGs; Table S11: Gene ontology (GO) analysis for all 384 common DEGs; Table S12: Gene ontology (GO) analysis for 200 common elevated DEGs; Table S13: Gene ontology (GO) analysis for 183 common reduced genes; Table S14 Overlapping genes between post-cataract surgery DEGs and TGFB1 common DEGs and their expression; Table S15: Hypergeometric enrichment analysis; Table S16: Directionality concordance analysis; Table S17: List of overlapping genes between human PCO and common 384 DEGs and expression in FPKM; Data S1: RNA-seq preprocessing; Data S2: R script for differential expression and downstream analysis.

## Abbreviations

The following abbreviations are used in this manuscript:

TGF-β	Transforming Growth Factor-beta
EMT	Epithelial–Mesenchymal Transition
ASC	Anterior Subcapsular Cataract
DEGs	Differentially Expressed Genes
